# Heat Waves: A Bibliometric Analysis of Thermotherapy Research

**DOI:** 10.7759/cureus.65700

**Published:** 2024-07-29

**Authors:** I John Berlin, Jobin Jose, Resmi S, Priyadarsini G, Vinoj M N

**Affiliations:** 1 Department of Physics, Mar Thoma College, Tiruvalla, IND; 2 Department of Library Science, Marian College Kuttikkanam (Autonomous), Kuttikkanam, IND; 3 Department of Physics, Sree Narayana College for Women, Kollam, IND; 4 Department of Physics, St. Peter's College, Kolenchery, IND

**Keywords:** vosviewer, biblioshiny, bibliometric analysis, thermotherapy, heat therapy

## Abstract

This bibliometric study analyzes the evolving field of thermotherapy, a medical treatment that utilizes heat to treat various conditions, including cancer, by applying controlled temperatures to targeted tissues. Utilizing bibliographic data from the core collection of Web of Science and analysis software Biblioshiny and VOSviewer, we analyzed several key metrics to gain insights into the development and trends in thermotherapy research. The annual scientific production revealed a significant increase in publications over the past two decades, reflecting growing interest in this field. Analysis of the most relevant authors and sources highlighted key contributors and influential journals. Trend topics demonstrated a shift from early focus areas like hyperthermia and laser-induced thermotherapy to recent advancements involving nanoparticles and combination therapies. The thematic map provided insights into core, emerging, and niche areas within the research landscape. A historiograph traced the chronological development of significant publications, while the co-occurrence of keywords and bibliographic coupling of documents identified major themes and interconnections in the literature. International collaborations were mapped, showing the global nature of thermotherapy research. The study identified several research gaps, including the need for large-scale clinical trials, interdisciplinary approaches, and standardized treatment protocols. Practical implications suggest focusing on targeted delivery systems, expanding cancer research, and fostering collaborative projects to advance the field.

## Introduction and background

Thermotherapy, commonly known as heat therapy, is a healing treatment whereby heat is introduced to the body to relieve pain and enhance healing [1,2]. The treatment has been in practice for several 100 years in many forms, running from the most elementary hot compresses to absolutely intricate thermal devices. Stimulation of blood flow, relaxation of muscles, reduction of stiffness in joints, and good health regarding tissue are the primary principles applied by thermotherapy by introducing heat [3]. Being one of the non-invasive and effective techniques, thermotherapy finds a broad range of applications in hospitals and at home for the treatment of a host of conditions related to musculoskeletal pain, arthritis, and chronic injuries [4].

Heat has been used as a therapeutic agent since ancient times [5]. Hot baths and steam rooms were favorites among Greeks and Romans not because of their relaxing properties but likely for associated health benefits [6]. Again, hot compresses and moxibustion - the burning of mugwort to heat areas of the body - have been entrenched in traditional Chinese medicine [7]. These early practices provided the basis for modern thermotherapy techniques with the changing medical science and technology. Heat applied to the body brings about a number of physiological reactions that can lead to its healing properties [5]. When heat is applied to the skin, this leads to vasodilation or the broadening of the blood vessels [2]. It promotes oxygen and nutrient delivery in addition to the removal of cellular waste products by increasing blood flow [3]. In turn, it improves the rate of healing and reduces inflammation. The brain, too, is actually directly impacted by heat. This can be due to the activation of heat-sensitive neurons that would lead to reducing the transmission of pain signals from a site of injury to the brain by inhibiting pain pathways [4]. This phenomenon, which is referred to as the gate control theory of pain, could explain the immediate analgesic effect of heat. In addition, the application of heat makes collagen tissues more flexible, which form part of muscles, tendons, and ligaments. This attribute is of particular benefit in situations where there is joint stiffness and muscle tightness due to the improvement in flexibility and range of motion [6].

There are numerous methods to transfer heat, each suited for different situations and personal preferences. Some common methods include dry heat therapy, moist heat therapy, infrared heat therapy, paraffin wax therapy, and fluid therapy [8,9]. Dry heat therapy uses heating pads, electric blankets, and heat lamps. Moist heat sources penetrate deeper into tissues and include hot baths and steamed towels to ensure good relaxation of muscles [10,11]. Infrared therapy uses lamps or saunas, whereby more deeply located skin layers get heated to treat deep tissue pathologies for chronic conditions like arthritis [12]. Paraffin wax therapy consists of hand or foot dips in warm wax that forms a warm, insulating layer beneficial in conditions such as rheumatoid arthritis. Fluid therapy applied to heat comprises generally fine cellulose dust that, blown by heated air, provides uniform heat and massage-like effects [8].

Thermotherapy has a variety of benefits, which prove to be helpful in the treatment of many conditions. First is pain relief because heat improves blood circulation and relaxes muscles that are usually responsible for producing pain [2,13]. Improved healing is also encouraged because, with better circulation resulting from applied heat, more oxygenated blood is delivered with nutrients to damaged tissues [13,14]. It relaxes tight and strained muscles, hence reducing spasms and increasing flexibility. Therefore, this shall be very useful for people suffering from chronic muscle tension or those who have undergone strenuous physical activity. Heat improves the extensibility of collagen tissues; therefore, it increases flexibility at the joints and the range of motion [13]. This increase in flexibility is quite significant in situations such as arthritis or even people suffering from joint stiffness [15]. Heat therapy has even psychological effects, as warmth and relaxation decrease stress and increase well-being. Heat therapy's easy, non-invasive nature allows it to be quickly administered at home or in a clinic [16]. It is, therefore, a safe and effective alternative to medication in the treatment of pain and improvement of healing.

Bibliometric analysis can provide a quantitative approach to measuring the scope and impact of the research in a given domain [17,18]. In the case of thermotherapy, this kind of analysis will be able to showcase the development of research, outline its significant contributors, and emphasize highly influential publications that have set a direction in the area under study. In that respect, the present study intends to underline a comprehensive bibliometric analysis of research in thermotherapy, mapping progress over time, collaborative networks, and emerging trends and gaps in research. Bibliometric indicators will be used, among others, to analyze the number of publications, citation analysis, patterns of authorship, and geographical distribution of research output [19-21]. This should provide an overall description of the actual situation regarding the research in thermotherapy and further yield strategic insights into where future investigations should be placed. A total of two powerful bibliometric analysis tools, Biblioshiny and VOSviewer, are used to systematically investigate the literature available on the theme of thermotherapy. Biblioshiny is an online application designed for bibliometric and scientometric analysis that allows the intuitive visualization of bibliometric data to provide full-scale Norwegian analysis, including citation, co-citation, and thematic mapping [22-24]. In contrast, VOSviewer specializes in the construction and visualization of bibliometric networks with co-authorship, co-occurrence, and citation network generation status, hence giving an in-depth insight into the structural relationship between research communities [25-27].

The objectives of this study are to analyze publication trends over time to describe the temporal distribution and patterns in thermotherapy research, identify major authors and leading journals by determining the most prolific contributors and influential journals in the field, and investigate the geographical distribution of research to pinpoint which countries and regions are leading in thermotherapy research while assessing the impact of international collaboration. Additionally, the study aims to identify key research themes and topics through keyword and topic analysis to reveal major areas of focus and emerging trends, analyze citation patterns to understand the impact and influence of thermotherapy research, including identifying highly cited papers and citation networks, and finally, to identify research gaps and future directions through a review of the available literature, providing insight into under-researched areas and offering recommendations for future studies.

## Review

Materials and methods

This bibliometric analysis was performed in the field of thermotherapy research using Web of Science's core collection, which is well-known for its extensive coverage and comprehensive compilation of peer-reviewed literature [28]. In the search query, "Thermotherapy" and "Heat therapy" were used to capture international breaths about thermotherapy research with no restriction on language for a complete investigation of this domain. In this work, only research articles were considered. Thus, studies were targeted as original research contributions to the field, excluding certain types of research from reviews, editorials, and other non-research articles. This result led to a return of 3425 documents relevant for analysis in this initial search. Metadata, including titles, authors, abstracts, keywords, years of publication, and citations, were retrieved and saved in plaintext files for later importation into Biblioshiny and VOSviewer to create a detailed bibliometric study.

Main Information of the Investigation

Table 1 provides a comprehensive overview of the bibliometric data on thermotherapy research from 1989 to 2024. The analysis covers publications from 1989 to 2024, indicating a broad temporal scope with contributions from 1086 sources, including journals and books, reflecting a substantial body of 3425 documents. The field exhibits an annual growth rate of 6.88%, suggesting a steady increase in research output, with an average document age of 13.5 years, mixing historical and recent studies. Each document receives an average of 27.17 citations, highlighting significant academic impact, and the total number of references cited is 75848, demonstrating extensive scholarly engagement. The document contents include 5676 keywords plus terms and 6452 unique author's keywords, indicating diverse topics within thermotherapy. The research involves 12972 authors, showing high collaboration, with only 121 single-authored documents, underscoring the collaborative nature of the field. On average, each document has 5.65 co-authors and 17.72% of the documents involve international collaboration, highlighting global interest and cooperation. Most of the documents (3404) are research articles, with 21 early access articles indicating some research is available before formal publication. Overall, the bibliometric analysis reveals that thermotherapy is a well-established and growing field of research with significant academic collaboration and impact.

**Table 1:** Main information of the investigation

Description	Results
Main information about data	
Timespan	1989:2024
Sources (journals, books, etc.)	1086
Documents	3425
Annual growth rate %	6.88
Document average age	13.5
Average citations per doc	27.17
References	75848
Document contents	
Keywords plus (ID)	5676
Author's keywords (DE)	6452
Authors	
Authors	12972
Authors of single-authored docs	121
Authors collaboration	
Single-authored docs	139
Co-authors per doc	5.65
International co-authorships %	17.72
Document types	
article	3404
article; early access	21

Findings and discussions

Annual Scientific Production

**Figure 1:**
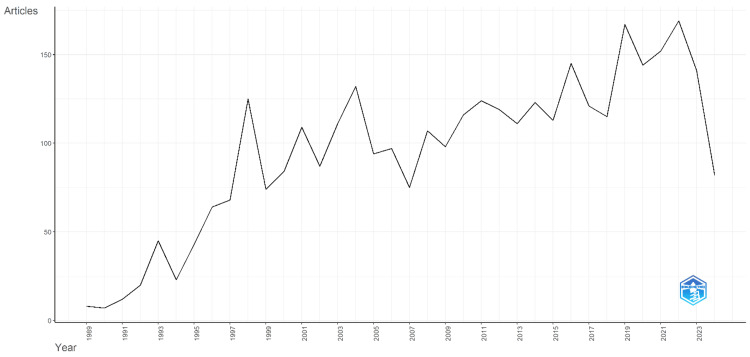
Annual scientific production from 1989 to 2024

Figure 1 depicts a clear upward trend in annual scientific production in thermotherapy research from 1989 to 2024. Starting with a modest eight articles in 1989, the number of publications gradually increased, with a notable surge in the mid-1990s. By 1998, the number of articles jumped to 125, indicating growing interest and research activity in the field. The early 2000s maintained this upward trajectory, with annual publications consistently surpassing 100 articles. Notably, 2022 peaked with 169 articles, reflecting heightened research output. Although the number for 2024 is lower at 82 articles, it is crucial to consider that this count only extends until April, suggesting that the full year’s data might still reflect a continuing robust research interest in thermotherapy. This trend underscores thermotherapy's increasing recognition and importance in scientific research, demonstrating a sustained and expanding academic focus over the past three decades.

Most Relevant Authors

**Figure 2:**
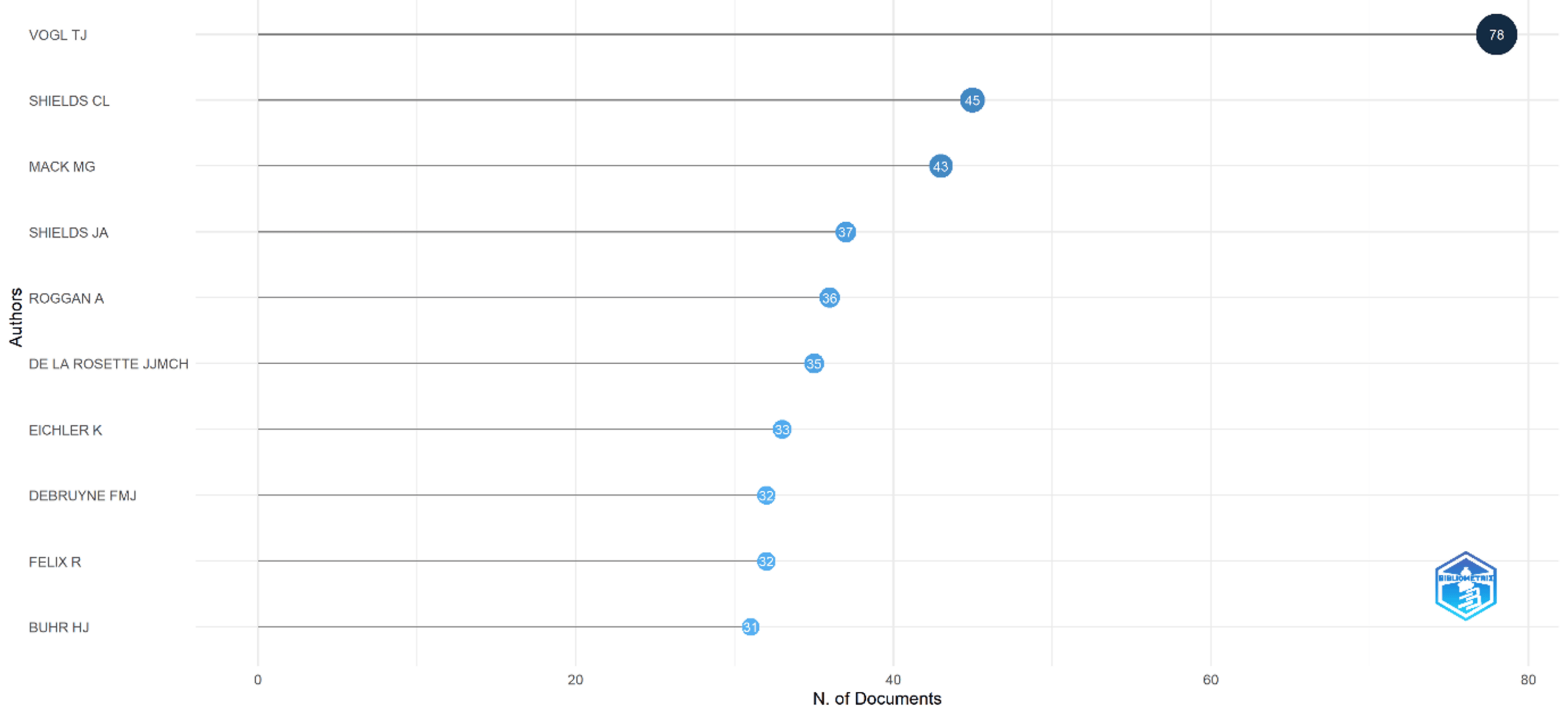
Most relevant authors

Figure 2 shows the authors who have been the most productive in thermotherapy research based on the number of published documents. Vogl TJ is the most prolific with 78 publications, followed by Shields CL with 45 documents and Mack MG with 43 documents. Other significant contributors are Shields JA with 37 publications, Roggan A with 36, and De La Rosette JJMC with 35 publications. The list includes Eichler K (33 documents), Debruyne FMJ (32 documents), Felix R (32 documents), and Buhr HJ (31 documents). This data highlights the substantial contributions of these authors to the field of thermotherapy research, with Vogl TJ leading by a significant margin.

Most Relevant Sources

Table 2 displays the key sources for publications in thermotherapy, emphasizing the leading journals and their impact on the number of published articles. On the top of this list is the *International Journal of Hyperthermia*, with 97 articles, therefore dominating the publication of literature on thermotherapy. This is followed by *Lasers in Surgery and Medicine*, which has 71 articles, thereby showing a solid relevance for laser therapeutic methods. Similarly, 64 articles have been published in *Urology *and 56 in the J*ournal of Urology*; thus, it can be inferred that there is strong support from the urology fraternity. The *Journal of Endourology* contains 53 articles, while 50 are found in *European Urology*, thus further elucidating the role of thermotherapy in urological treatment. *Lasers in Medical Science* has 42 articles, reflecting its emphasis on medical laser applications. *BJU International* (36 articles) and *Retina-The Journal of Retinal and Vitreous Diseases* (35 articles) indicate the application of thermotherapy in both urology and ophthalmology. Last, *ACS Applied Materials & Interfaces*, with 32 articles, suggests a focus on the material science aspects of thermotherapy. This distribution of articles across various journals underscores the multidisciplinary nature of thermotherapy research, spanning across hyperthermia treatments, surgical applications, and material sciences.

**Table 2:** Most relevant sources

Sources	Articles
International Journal of Hyperthermia	97
Lasers in Surgery and Medicine	71
Urology	64
Journal of Urology	56
Journal of Endourology	53
European Urology	50
Lasers in Medical Science	42
BJU International	36
Retina-The Journal of Retinal and Vitreous Diseases	35
ACS Applied Materials & Interfaces	32

Countries' Scientific Production

Table 3 presents the scientific production in thermotherapy research by country. The USA leads significantly with 2029 documents, indicating its significant investment and research activity. China follows with 1417 documents, indicating a strong and growing focus on thermotherapy. Germany also shows substantial contributions with 1132 documents. Other notable contributors include France (404 documents) and Italy (398 documents), demonstrating considerable research output in Europe. Japan has produced 348 documents, showing significant engagement from Asia, while South Korea and the UK each have 320 documents, highlighting their involvement in the global research landscape. The Netherlands (258 documents) and Canada (249 documents) also contribute meaningfully, underscoring the global nature of thermotherapy research with significant contributions from North America, Europe, and Asia.

**Table 3:** Countries’ scientific production

Region	Number of documents
USA	2029
China	1417
Germany	1132
France	404
Italy	398
Japan	348
South Korea	320
UK	320
Netherlands	258
Canada	249

Historiograph

**Figure 3:**
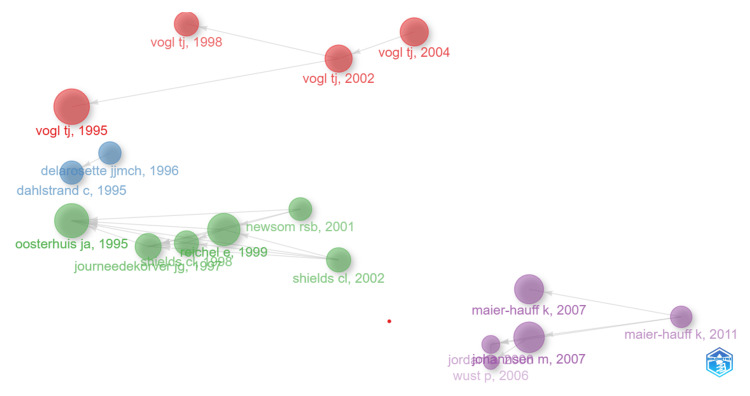
Historical development and evolution of thermotherapy research

The historiograph visualizes how the citation relationships among key thermotherapy research publications looked like over time. This graph thus expresses how different authors and their works are connected by citations executed, putting the created influences and impacts in relief within particular studies (Figure 3).

Clusters and influential authors: It vividly indicates clear clusters of influential authors. Major ones in the red cluster include Vogl TJ. Significant works from this author were done in 1995, 1998, 2002, and 2004. In his recent article "Interventional treatments of colorectal liver metastases using thermal ablation and transarterial chemoembolization: a single-center experience over 26 years," the author highlights the scarcity of long-term studies on locoregional treatments for colorectal liver metastases (CRLM), despite the frequent use of methods like laser-induced thermotherapy, microwave ablation, and transarterial chemoembolization. He presents a comprehensive retrospective study involving 2140 patients over 26 years, demonstrating the varying efficacy and survival outcomes of these treatments [29]. From this pattern, it becomes clear that Vogl TJ stands among one of the central contributors whose works are central in representing basic working materials and are widely referred to in the area under study. The Green Cluster consists of highly associated authors such as "Oosterhuis JA" from 1995 and subsequent Gä "JourneedeKrover PJ" in 1997; "Reichel E" in 1999; and "Shields CL" in 2002. These four publications are considered interrelated. From this group of highlighted authors, one could presume, at worst, that the works are of a research collaborative or PŘ/study on similar themes. In the case of the purple cluster, key contributing authors identified include preeminently "Maier-Hauff K" in 2007 and 2011. Other influential publications in the cluster are works by "Johannsen M" from 2007 and "Wust P" from 2006, building a network of connected research activities.

Temporal evolution: The publication timeline is inaugurated by seminal works published in 1995 by "Oosterhuis JA" and "Dahlstrand C." This is followed by some critical publications of "Vogl TJ" ranging from 1995 to 2004, which indicates that the author was involved with sundry publications within a very short period. The green cluster indicates some continuity of research during the late 1990s, stretching into the early 2000s, while the purple cluster contributes some more recent vital pieces of research, especially around 2007 and 2011.

Citation links: The edges between different nodes, namely publications, are given to identify their citation relationship. In another way, this tells how more recent research has utilized or references earlier research. The thickness and size of lines and nodes identify the frequency and impact of said citations. A larger node and thicker lines indicate more highly cited work, hence significantly placing more emphasis on its potential impact within the circle of research.

Research focus: The separation between clusters may suggest distinct research themes or areas within thermotherapy. For instance, the works by "Vogl TJ" may focus on different aspects or applications of thermotherapy compared to those by "Maier-Hauff K." This differentiation in focus can help identify the various subfields and specialized topics that have emerged within the broader domain of thermotherapy research.

Trend Topics

**Figure 4:**
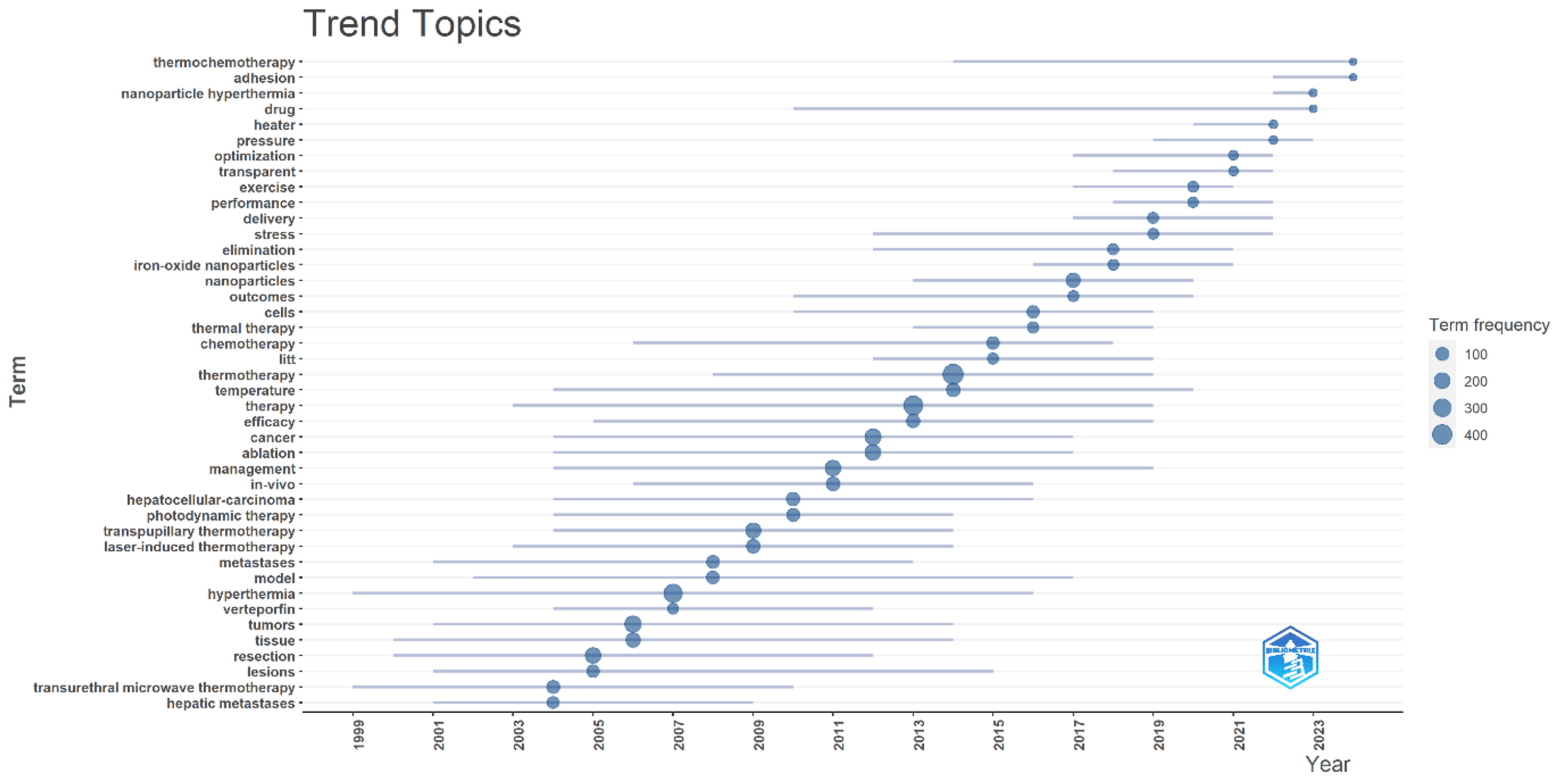
Trend topics

Figure 4 shows trend topics that visualize the evolution and frequency of various terms in thermotherapy research over the past two decades. The graph shows a clear upward trend in the frequency of terms associated with thermotherapy research, indicating growing interest and research output in this field. The term frequency is represented through the size of the circles; the more significant the circle, the higher the frequency. Terms prominent in the early 2000s were "hyperthermia," "laser-induced thermotherapy," and "transurethral microwave thermotherapy." These terms had been concentrated at the beginning stage of research related to these areas. Indeed, as the field worked its way through the middle 2000s toward the early 2010s, several leading terms - such as "temperature," "efficacy," "therapy," and "management" - came forward, denoting a shift toward evaluating effectivity and management strategies for thermotherapy. It was during this period that "hepatocellular carcinoma" and "hepatic metastases" were also hegemonically researched, clearly attesting to the fact that investigations have been focusing on treatment methods against liver cancers. From 2010 to the present, emerging terms like "nanoparticles," "iron-oxide nanoparticles," and "nanoparticle hyperthermia" show that explorations have oriented themselves toward advanced materials and techniques in thermotherapy. The high frequency of the terms "cancer," "chemotherapy," and "ablation" shows that this technology is being studied for use in cancer treatment applications. In recent years, high-frequency terms are "nanoparticle hyperthermia," "iron-oxide nanoparticles," and "thermochemotherapy," which point to cutting-edge research involving nanotechnology and combined treatment modality. The terms "delivery" and "optimization" come up frequently, indicating that the search for improvement in the precision and efficacy of thermotherapy treatments is continuous. While the focus has changed over the years from general hyperthermia techniques to more specific, advanced applications involving nanoparticles, combination therapies, and so on, cancer treatment has been the constant factor. The terminological diversity within a domain like "chemotherapy," "nanoparticles," and "exercise" supposes the cooperation or, better, interdisciplinarity of researchers working on thermotherapy, reflecting thus the dynamics and evolution of the field in question.

Thematic Map

**Figure 5:**
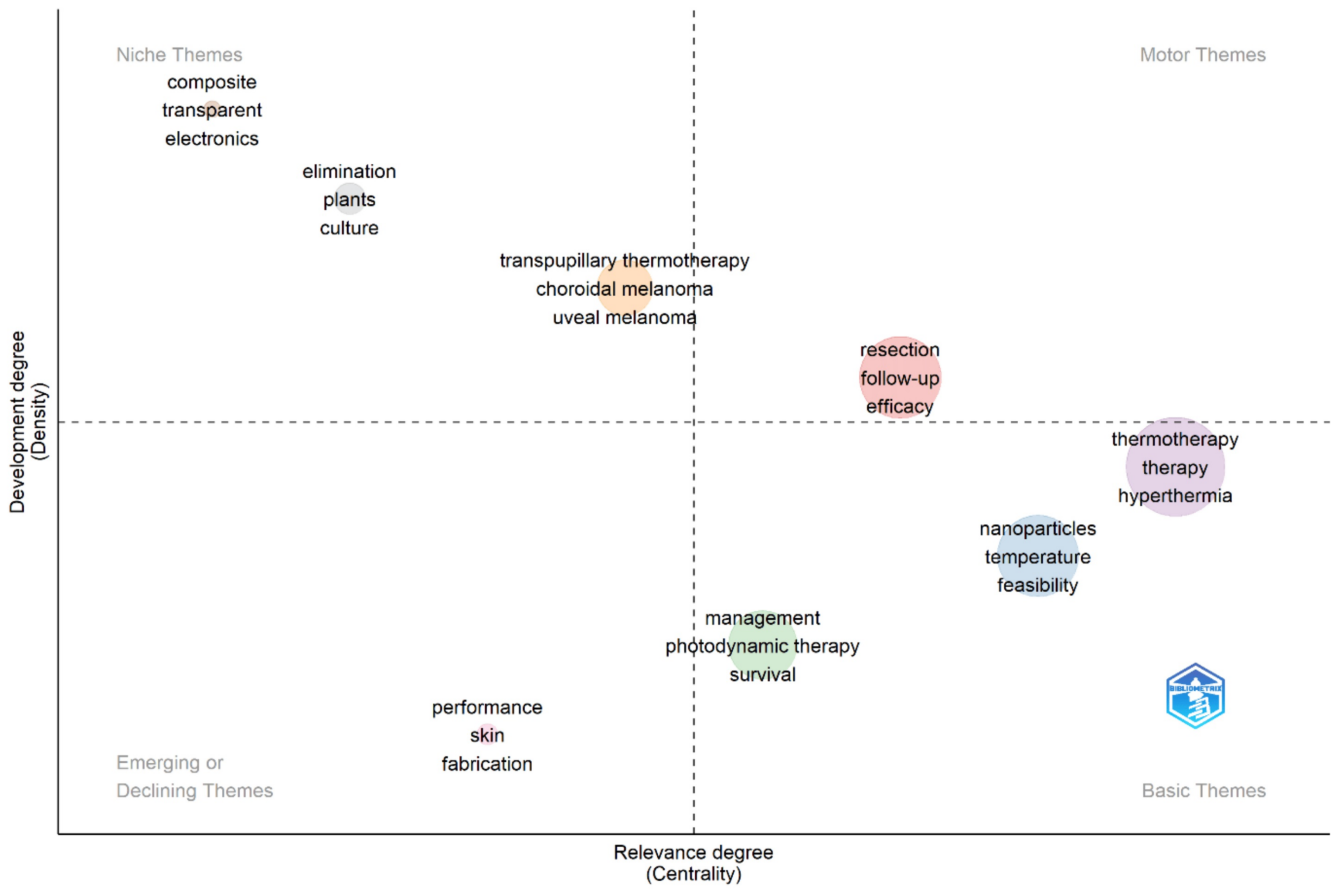
Thematic visualization of keywords

Figure 5 represents a "landscape" of the themes in thermotherapy research based on their relevance and degree of development. Therefore, in the upper-right quadrant, so-called motor themes such as "Thermotherapy," "Therapy," and "Hyperthermia" are high in development and high in relevance for the field simultaneously. These terms indicate central topics that are crucial and well-established in thermotherapy research, reflecting core areas of investigation and application. "Hyperthermia" refers to the therapeutic use of heat to treat various medical conditions, including cancer. When combined with radiation therapy, hyperthermia enhances the effectiveness of the treatment by raising the temperature of tumor tissues to between 40°C and 45°C (104-113°F). This increase in temperature makes cancer cells more sensitive to radiation by disrupting their structure and inhibiting their ability to repair radiation-induced damage. Additionally, hyperthermia improves blood flow to the tumor, enhancing oxygen delivery and making the tumor environment more susceptible to radiation. This synergistic effect can lead to better tumor control and improved survival rates in patients, highlighting the importance of hyperthermia in cancer treatment. The upper-left quadrant includes well-developed niche themes of lower relevance to the general field: "Composite," "Transparent," "Electronics," "Elimination," "Plants," and "Culture." These terms stand for specialized areas, well developed but having a marginal position about the central stream of research into thermotherapy. They could concern subfields or interdisciplinary applications. On the lower-right quadrant, basic themes like "Nanoparticles," "Temperature," and "Feasibility" are fundamental and relevant but not yet highly developed. These words underline essential areas of research where basic studies are going on, which may be essential to this discipline and may establish and integrate into mainstream research subsequently. Other important growing areas in the domain of thermotherapy are represented by terms such as "Management," "Photodynamic Therapy," and "Survival." The lower-left quadrant includes terms such as "Performance," "Skin," and "Fabrication," which constitute emerging areas in the very early stages of research or declining topics losing interest in the field. Specific clusters, like "Resection," "Follow-up, and "Efficacy," are strongly related to thematic areas like clinical outcomes and treatment effectiveness; others, like "Transpupillary Thermotherapy," "Choroidal Melanoma," and "Uveal Melanoma," are relevant but in need of further development. Such thematic mapping gives a strategic view of the state of the art about research in thermotherapy under examination, in this case, mature and evolving areas within the field.

Bibliographic Coupling of Documents

**Figure 6:**
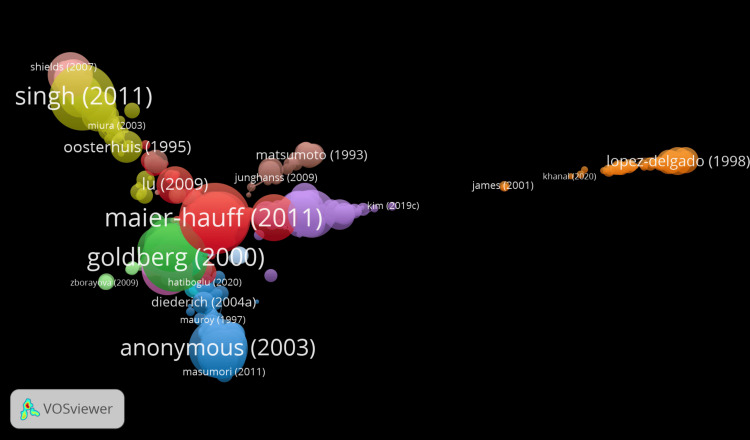
Bibliographic coupling of documents

Figure 6 displays a network visualization of bibliographic coupling used to map the scholarly discourse in the field of thermotherapy. This network encompasses 3425 documents, of which 2514 meet the citation criteria, resulting in a network of 2451 items distributed across 13 distinct clusters. Each cluster represents a thematic grouping of documents, with cluster size and prominent authors offering insights into focal research areas and influential contributors. Cluster 1 (red), with 477 items, likely represents foundational research featuring authors like Maier-Hauff (2011) and Goldberg (2000). Cluster 2 (green) includes 360 items and focuses on significant sub-disciplines, with Singh (2011) and Oosterhuis (1995) as key authors. Cluster 3 (blue) has 355 items, suggesting substantial work with authors such as Anonymous (2003) and Zborayova (2009). Cluster 4 (yellow) has 310 items, and notable authors include Shields (2007) and Miura (2003). Cluster 5 (purple) contains 269 items, featuring Matsumoto (1993) and Junghanss (2009). Cluster 6 (light blue) has 233 items, with authors like Diederich (2004a) and Hatiboglu (2020). Cluster 7 (orange) includes 177 items, with Lopez-Delgado (1998) and James (2001) as key contributors. Cluster 8 (brown) has 88 items, featuring Kim (2019c) and Khanal (2020). Cluster 9 (pink) contains 86 items, while cluster 10 (light green) has 48 items, cluster 11 (light blue) has 34 items, cluster 12 (light yellow) has 10 items, and cluster 13 (yellow) has 4 items, with prominent authors in these smaller clusters yet to be determined. This visualization highlights the extensive and varied research landscape in thermotherapy. It provides insights into focus areas and influential research through cluster sizes and prominent authors.

Co-occurrence of All Keywords

**Figure 7:**
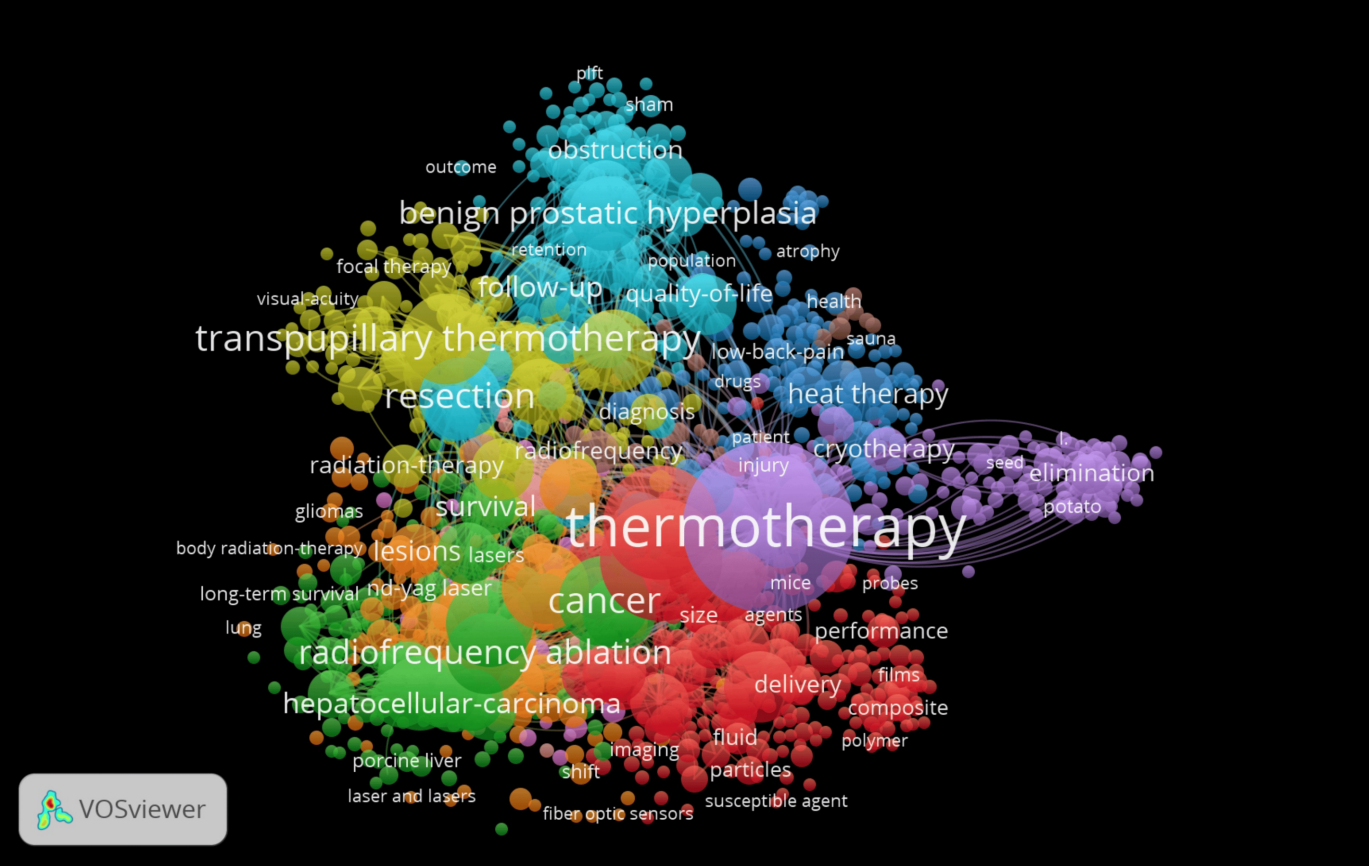
Co-occurrence of all keywords

The co-occurrence network shown in Figure 7 visualizes the relationships among 1126 keywords in the field of thermotherapy. These keywords are organized into 10 distinct clusters out of a total of 11078 keywords that have a minimum occurrence threshold of 5. This visualization provides insights into the thematic structure of the field. Cluster 1 (red) focuses on core techniques and applications of thermotherapy in cancer treatment, including advancements in material science. Cluster 2 (green) centers around radiation therapy and its impact on survival outcomes, particularly in liver and lung cancers. Cluster 3 (blue) highlights specific procedures like transpupillary thermotherapy and resection in the context of benign prostatic hyperplasia and ocular applications. Cluster 4 (yellow) explores broader applications of heat and cryotherapy, focusing on pain management and quality of life improvements. Cluster 5 (purple) emphasizes diagnostic techniques and imaging technologies, particularly lasers and radiofrequency. Cluster 6 (light blue) addresses outcomes and health impacts in large population studies. Cluster 7 (orange) highlights research on gliomas and the effects of body radiation therapy on survival rates. Cluster 8 (brown) likely focuses on follow-up and retention in clinical studies. Cluster 9 (pink) concentrates on experimental studies using mice. Cluster 10 (light pink) appears to focus on agricultural or environmental applications of thermotherapy.

Co-authorship of Countries

**Figure 8:**
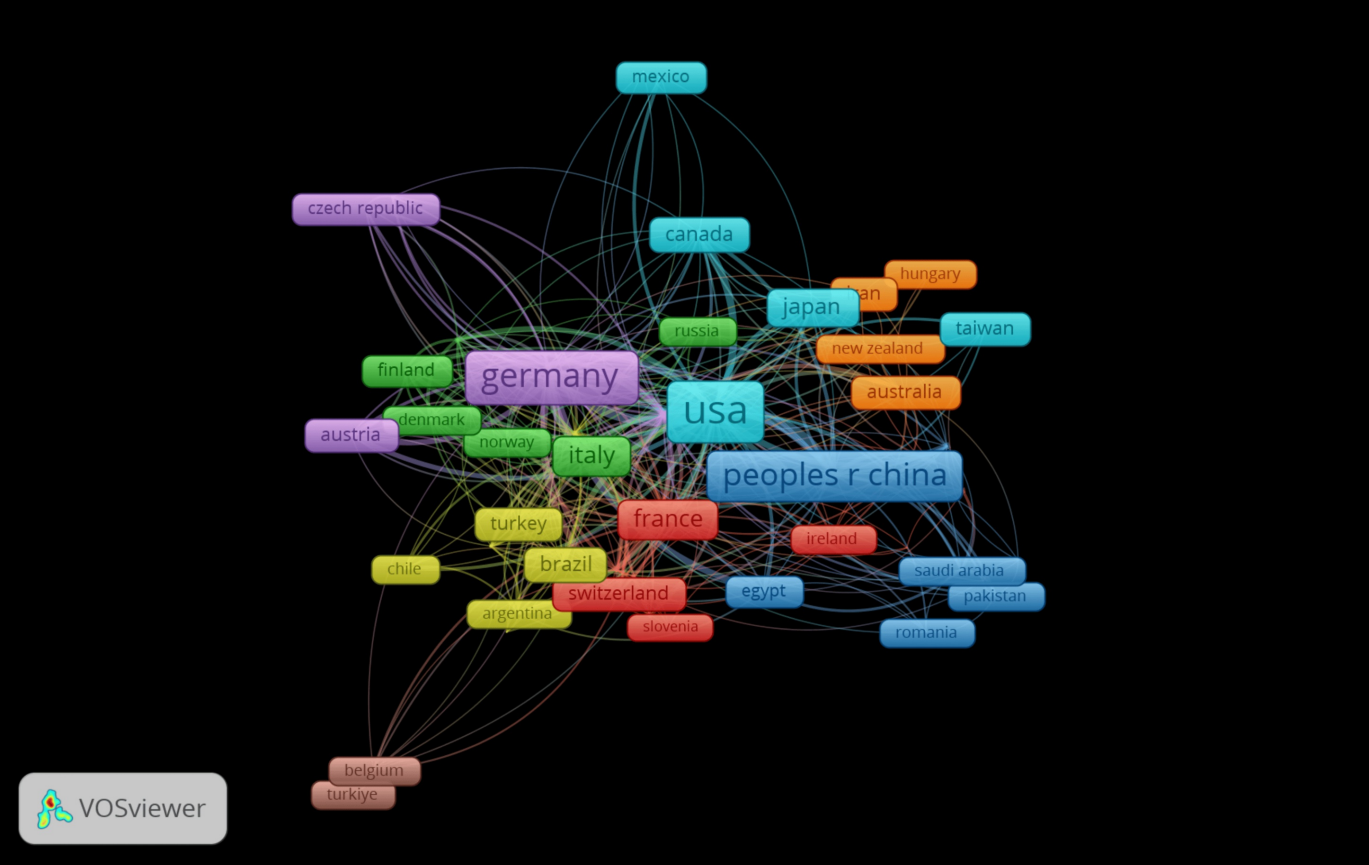
Co-authorship of countries

Figure 8 illustrates the network visualization of co-authorship countries in thermotherapy research, revealing the international collaboration patterns. The analysis includes 104 countries, with 51 meeting the minimum threshold of 5 documents, organized into 8 distinct clusters. Cluster 1 (red) highlights collaboration primarily among European countries and Turkey, featuring France, Turkey, and Belgium. Cluster 2 (green) focuses on collaborations between Southern European and Latin American countries, including Italy, Brazil, and Argentina. Cluster 3 (blue) represents North American collaboration with the USA as a central node alongside Canada and Mexico. Cluster 4 (yellow) highlights cooperation among German-speaking countries and neighboring regions such as Germany, Switzerland, and Austria. Cluster 5 (purple) is centered around Nordic collaborations, including Finland, Denmark, and Norway. Cluster 6 (light blue) focuses on East Asian countries like Japan, South Korea, and Taiwan. Cluster 7 (orange) represents a collaboration between the Pacific region and Hungary, featuring Australia, New Zealand, and Hungary. Cluster 8 (brown) highlights specific collaborations within the Middle East, including Saudi Arabia and Egypt. The network visualization demonstrates extensive international collaboration in thermotherapy research, with significant regional clusters reflecting geographical and cultural proximities.

Research gaps and practical implications

Interest in advanced nanomaterials and interdisciplinarity has been growing in thermotherapy, but significant gaps remain. For example, mainstream clinical practice integration of nanoparticles, like iron-oxide nanoparticles, has still not occurred due to a lack of large-scale clinical trials about their safety and efficacy. Finally, while cross-discipline collaboration could be possible, very little is known about its potential. Such interdisciplinary research can open up newer modalities of treatment that help optimize patient outcomes. Moreover, accurate and controlled delivery modes for thermotherapy agents are still under development. Research aimed in this direction could develop target-oriented delivery systems that make specific minimal damage occur to the surrounding healthy tissue. Standardized protocols for evaluating the efficacy of thermotherapy are also lacking, thus accounting for variable outcomes seen in clinical settings. They are emerging research areas in performance, skin, and fabrication that demand constant vigilance and review to capture new opportunities and allow for the redeployment of resources. Last, the treatment of cancer is one sizeable central part that indicates more comprehensive studies are needed to show the effectiveness of thermotherapy across ranges of types and stages of cancers to unleash its full potential, if any, and chart better clinics.

The following practical steps regarding thermotherapy could be advanced to help address these gaps: the design and performance of prospective, randomized controlled trials with large sample sizes and multi-center settings to validate efficacy and safety in advanced techniques, especially nanotechnology; stimulating collaborative research projects within different disciplines, like oncology, materials science, and biomedical engineering; and developing new, multifaceted treatment approaches. Development and testing of targeted delivery systems regarding thermotherapy agents will increase precision, reduce side effects, and improve patient safety. It is fruitful that standardization of treatment protocols through collaboration with regulatory bodies ensures homogeneous and reliable results. The research community could, therefore, remain agile and responsive to new developments by regularly reviewing and analyzing emerging trends in research to identify new opportunities. This must also include the extension of cancer research that has not so far been extended to lesser and rare forms, optimal usage of thermotherapy in oncology, and the discovery of new indications for improvement in the treatment of patients. Through these practical steps and filling these gaps, a great leap can happen in thermotherapy research, significantly improving patients' chances of better treatment options and results.

To strengthen the connection between the bibliometric findings and their practical implications for researchers and practitioners in thermotherapy, it is essential to highlight specific examples of how identified trends and gaps can guide future research directions. For instance, the increasing focus on nanotechnology in thermotherapy, as evidenced by frequent terms like "nanoparticles" and "iron-oxide nanoparticles," suggests that future research should prioritize large-scale clinical trials to establish the safety and efficacy of these advanced materials in clinical practice. Researchers could also explore interdisciplinary collaborations, combining expertise in oncology, materials science, and biomedical engineering, to develop innovative thermotherapy techniques that enhance precision and minimize side effects. The observed gaps in standardized treatment protocols highlight the need for establishing uniform guidelines through collaborative efforts with regulatory bodies, ensuring consistent and reliable outcomes across different clinical settings. Moreover, the emerging interest in areas such as "management," "photodynamic therapy," and "survival" indicates that future studies should investigate the integration of thermotherapy with other treatment modalities to optimize patient outcomes. By addressing these specific examples, researchers and practitioners can better align their efforts with the evolving landscape of thermotherapy research, ultimately advancing the field and improving therapeutic options for patients.

This bibliometric analysis relies exclusively on data from the Web of Science (WoS), which, while extensive, may introduce certain biases. WoS primarily includes peer-reviewed journals, potentially underrepresenting other important sources like conference proceedings, theses, and other indexed journals. This reliance on a single database may result in a skewed view of the research landscape. Additionally, bibliometric methods primarily focus on quantitative metrics such as citation counts, which may not fully capture the qualitative aspects of research impact, such as the significance of findings or contributions to practice.

## Conclusions

This bibliometric study on thermotherapy shows an area of fast growth with changing trends, actually reflecting increased research interest and, therefore, development. Significant contributors, influential journals, and central themes were detected, portraying the global, interdisciplinary dimensions of thermotherapy research. The other trend shift is toward advanced nanomaterials and combination therapies, mainly for cancer therapy. Some noted research gaps include further randomized controlled trials, delivery methods, and standardized treatment protocols. Large, multi-center randomized controlled trials are therefore indicated to further validate the safety and efficacy of the emerging thermotherapy techniques. Encouraging interdisciplinary collaborative efforts could yield combined innovative treatment modalities that will improve patient outcomes. The second aspect is that developing precise and controlled formulations for thermotherapy agents will bring accuracy to the treatment and reduce side effects. Addressing these gaps and implementing the recommendations outlined in this report will enable thermotherapy to reach its full potential as a versatile and effective treatment modality.
